# Children's Headache: The Difference Between Children's and Mothers' Perceptions and Awareness

**DOI:** 10.1155/prm/7024598

**Published:** 2025-10-26

**Authors:** Salvatore Lo Cascio, Edvige Correnti, Federica Cernigliaro, Floriana Ferro, Giuseppe Santangelo, Vittorio Sciruicchio, Filippo Brighina, Vincenzo Raieli

**Affiliations:** ^1^Child Neuropsychiatry Unit Department, PROMISE “G. D'Alessandro”, University of Palermo, Palermo, Italy; ^2^Child Neuropsychiatry Department, ISMEP, ARNAS Civico, Palermo, Italy; ^3^Children Epilepsy and EEG Center, PO, San Paolo ASL, Bari, Italy; ^4^Department of Biomedicine, Neuroscience and Advanced Diagnostic, University of Palermo, Palermo, Italy

**Keywords:** children, headache, ID migraine, migraine, mothers

## Abstract

**Background:**

In childhood, assessing the history of headaches in children can be challenging due to their age and the reliance on parental reports, which may sometimes lead to under/over-reporting of symptoms. This study utilized the ID Migraine questionnaire to evaluate the presence of headaches in a sample of children and examined the correlation between the children's responses and those of their mothers.

**Methods:**

A cohort of children aged 10–12 years and their mothers administered the ID Migraine questionnaire separately, which included questions about the children's headaches.

**Results:**

The cohort comprised 68 children (35 females and 33 males) aged 10–12 years. Within the last 3 months, 24/68 (35.3%) reported experiencing a history of headache episodes. Headache was reported by 16/35 females (45.7%) and 8/33 males (24.2%). Of the total, only 2/68 (2.95%) met two of the three criteria for potential migraine classification, 11/68 (16.1%) met at least one criterion, and 11 reported headaches but did not meet any criteria. Discrepancies between the children's and mother's responses occurred in 23/68 cases (33.8%), of which 14 (about 61%) disagreed about the occurrence of headaches in the past 3 months and 9 (about 49%) had inconsistencies in their responses to the Migraine ID items.

**Conclusions:**

These results highlight the challenges of collecting headache data in children under 12 years of age. Notably, there is a discrepancy between children's and mothers' reports, with underreporting headaches reported by the other in nearly 60% of the conflicting cases. Additionally, the incidence of migraine observed in this sample was lower than what is commonly suggested in the literature.

## 1. Introduction

In infants and children, understanding the medical history of headaches, both epidemiologically and clinically, can be challenging for two key reasons: the child's age and the need to gather information from parents, who may sometimes underreport, not report, or not observe. In children under 7 years, the primary source of medical history is usually the mother, who also plays a vital role in establishing the foundational “doctor–patient alliance.” However, in adolescents, clinicians must focus primarily on the adolescent's own narrative and needs, which often differ from those of the mother [1]. For children aged 7–12, clinicians must consider both perspectives (the child's and the mother's) and integrate them into a coherent framework. Yet, collecting information from two distinct sources can introduce additional challenges [2].

These diagnostic challenges should be recognized and addressed by pediatric headache specialists, but they have received limited attention from the scientific community. The few studies available do not refute the existence of these challenges and risks in managing pediatric headaches; rather, they provide some insight into the issue [3–8]. For example, Antoniuk et al., 1998 [3] reported only 40% agreement between children aged 10–14 years and their parents on frequency of unspecified headache types. In a study of 2601 children with an average age of 10 years, Sasmaz et al., 2004 [4] found that about 25% of the parents were unaware of their children's headaches (including migraine or tension headache or not classified headache), with no significant difference according to social background. Subsequently, Lundquist et al., 2006 [5] observed that the prevalence of primary headache (not distinguished between migraine and tension-type headache) in girls aged 7–12 years was underestimated, especially by fathers. Similarly, Nakamura et al., 2013 [6] reported a low correlation (0.39) between the presence of headache (especially migraine) and parental perception in a US population aged 13–18 years, and an even lower correlation in boys with headache.

In Italy, there is no available data on this issue. However, but the presence of a well-established pediatric care network—a distinctive feature of the Italian healthcare system—may help parents better understand their children's health needs, including headache-related problems. Although existing literature may not be fully up to date, it is worth noting that in recent years have seen heightened awareness of headaches, particularly migraines, due to increased attention from the mass media [9].

This rise in public awareness could potentially reduce discrepancies between children's and parents' perceptions of headaches. Given these factors, this study aims to evaluate the correlation in the perception of headache as a problem between children aged 10–12 years and their mothers.

The study is part of a broader project focused on improving the diagnostic process for neurological disorders in pediatric outpatient and emergency departments, as well as examining follow-up care. Our hypothesis was that there was an excellent agreement between the two news sources considering the simplicity and comprehensibility of the questionnaire used.

## 2. Methods

In the second half of 2021, we recruited a population of children aged 10–12 years who attended a primary care pediatric outpatient clinics for reasons unrelated to headache. Children with previously diagnosed primary headaches by their pediatrician were not included in the study. There were no additional inclusion criteria. However, we excluded cases where either the child or the mother had severe psychiatric disorders, severe neurological diseases, or intellectual disability.

The ID Migraine questionnaire was administered separately and independently to children and their mothers by nurses working in pediatric outpatient clinic.

The nurses gave the sheet to the child and the mother separately and asked them to answer the questions. The child reported about their own headaches, while the mother reported about the child's headaches.

Additionally, this group was used to test the potential usefulness of the Italian version of the ID Migraine questionnaire in the pediatric setting, similar to the validation performed in adults [10]. The latter aim was to facilitate rapid identification of patients and to assess the accuracy of pediatricians' perception of migraine.  Table 1 shows the questions of ID Migraine questionnaire. In this article, we present only the data obtained from a brief screening for the presence of headache using the ID Migraine in children attending the pediatric clinic for problems not related to headache. In this article, we present only the data obtained from a brief screening for the presence of headache using the ID Migraine in children attending the pediatric clinic for problems not related to headache.

Administering the ID Migraine questionnaire separately to mothers would have also helped assess the applicability of the test. The mothers' responses could provide additional confirmation of the presence and type of headache that the ID Migraine could detected in our pediatric sample.

We used Cohen's kappa to calculate the nonrandom agreement in addition to presenting the data descriptively.

The test was administered only after obtaining written informed consent from the mothers and following validation by the Ethics Committee (minutes no. 51; protocol number 263, Palermo Civic Hospital, on December 14, 2020).

Discrepancies between the child's and mother's responses occurred in 23/68 cases (33.8%) of the full sample, of which 14/23 (60.8%) of the disagreed responses were related to the onset of headache in the last 3 months and instead 9/23 (39.2%) disagreed in their responses to the ID items on migraine. The Cohen's Kappa: 0.45 (moderate agreement between the two samples of children and mothers on the presence of headache in the child in the last 3 months).  Table 2 shows the summarized data of our sample.

## 3. Results

Overall, discrepancies between child and mother responses were identified in 23 of 68 cases (33.8%). Of these disagreements, 14 (60.8%) concerned the presence or absence of headache in the past three months, while the remaining 9 (39.2%) related to responses on migraine-specific ID criteria.

The agreement between child and mother reports on the presence of headaches in the past 3 months was moderate, with a Cohen's kappa coefficient of 0.45.

Table 2 summarizes the detailed findings.

However, concordance between the child and mother responses was limited: in 14 of the 24 headache-positive cases (58.3%), the responses did not match.

The cohort consisted of 68 children (35 females and 33 males) aged 10–12 years, with a mean age of 10 ys, 7 mo. Based on questionnaire responses completed by both children and their mothers, 24 out of 68 children (35.3%) reported experiencing headache episodes within the last three months. Headaches were reported more frequently among females (16/35; 45.7%) compared with males (8/33; 24.2%).

Children self-reported headache in 19 out of 68 cases (27.9%), while mothers reported headaches in their children in 15 out of 68 cases (22.1%). Regarding migraine classification, only 2 children (2.95%) met two out of three criteria suggestive of a potential migraine diagnosis. An additional 11 children (16.1%) met at least one criterion, while 11 others reported headaches that did not fulfill any migraine criteria.

Overall, discrepancies between the child's and mother' responses were identified to have occurred in 23/68 cases (33.8%)

Of these disagreements, 14/23 (60.8%) concerned the presence or absence of headache in the last 3 months, while the remaining 9/23 (39.2%) disagreed in their responses to the ID items on migraine. The agreement between child and mother reports on the presence of headaches in the past 3 months was moderate, with a Cohen's kappa coefficient of 0.45.

Table 2 shows the summarized data of our sample.

## 4. Discussion

Our data confirm the limited literature on the discrepancy in perception of headache between children and parents, particularly in an age group where obtaining information from both sources is essential [3–8]. The few studies in the literature, although sometimes conducted with different methodologies and on pediatric populations with not entirely of the same age, report concordance rates ranging from 36.3% to 55% and up to 73.5%; sensitivity and specificity of parents' answers of 65% and 55% respectively; and a Cohen's Kappa coefficient of agreement between children and parents of 0.39. These results do not differ significantly from ours, which show a general disagreement rate of 33.8% and a Cohen's Kappa of 0.45.

In 14/68 (20,58%) in our sample, there is disagreement between children and mothers regarding the presence of headaches in the last three months, with discrepancies most often resulting from a positive endorsement of headache by the child but a report of no headache by the mother. This discrepancy becomes even more evident apparent when considering that our study used simple questions from the ID Migraine questionnaire.

The observation that mothers mainly do not report their children's headache episodes, rather than children failing to remember the attacks, may be due to parents underestimating the severity of headaches.

In our opinion, the sentence describes the objective data, while the last part offers a possible interpretation, without making any moral judgment [5]. This is particularly evident when the headaches are infrequent, not accompanied by neurological symptoms, and when some parents may be attuned to the fact that headaches may serve an avoidance function for their child (e.g., avoidance of school) or attributed to other nonmedical such as electronic devices [3, 7]. This finding aligns with our other study, which showed that mothers tend to underestimate their children's use of analgesics like acetaminophen, frequently administered for several days during febrile episodes [1].

Exploring how parents perceive their own pain experiences may also have impact on their observations of and attention to their child's pain. It would be valuable to administer a pain perception questionnaire to both groups. This could help determine whether a heightened focus on their pain by mothers or, conversely, a lack of attention to it, influences their attitudes toward their child's pain. Another interesting question is whether there are differences between mothers and fathers in perceiving their child's headaches. However, our study cannot address this, as it is typically mothers who accompany children—especially those under 12 years old—to outpatient clinics and emergency departments.

In southern Italy, mothers are usually the parents who accompany the child to the pediatrician or pediatric neurologist [11]. Fathers may sometimes accompany the child alongside the mother, but rarely do so alone. Moreover, mothers tend to be the most knowledgeable about their child's health and are responsible for managing the therapy [1], a pattern that has also been reported in other countries. [12, 13]. For this reason, reaching an adequate sample of male parents would have required significantly more time, and the involvement of additional caregivers could have confounded the evaluation of the data. Furthermore, this study was part of a larger project, and it was not necessary to involve fathers as well.

While Lundquist et al. [5] suggest this possibility, further studies are needed to confirm such findings. It would certainly be beneficial to design a study involving both mothers and fathers concurrently to compare their responses.

Furthermore, our findings highlight the importance for pediatricians to recognize that the expectations and experiences of children with headaches may not always align with those of their mothers, due to the differing perceptions of headache occurrence between the two. Additionally, the prevalence of migraine in our sample of children without a history of headaches appears lower than what is typically reported in the literature [9]. This discrepancy raises concerns about the reliability of the ID Migraine tool as a quick and effective screening method for suspected migraines in childhood, particularly in this age group, for assisting pediatricians or conducting epidemiological studies [14, 15]. Larger studies are certainly needed to confirm the validity of ID Migraine as a screening tool for pediatric migraines.

A recent study [16] on the validation of the Italian version of ID Migraine questionnaire reported excellent specificity and good sensitivity in a pediatric population aged 6–17 years. However, since the ID Migraine questionnaire was administered only to minors, it did not address the potential differences in responses between children and their mothers, which is a key focus of our study.

Lastly, it is also important to acknowledge that a larger study is necessary to confirm or refute our findings, given the limitations of our current research. One such limitation stems from the unexpected nature of the data collection, as the data were gathered during a study with a different primary objective. Additionally, our results may be influenced by cultural factors; however, a recent Canadian study [17] involving a similarly sized youth sample also found low agreement between parents and adolescents regarding headaches, reflecting what was observed in our study as well as in previous research.

Another potential limitation of our study is the possibility of retrospective memory bias concerning headache episodes reported by both the child and the mother. However, it is worth noting that the ID Migraine questionnaire focuses on a relatively short recall period, the last three months, which we believe reduces the likelihood of memory bias. This context underscores the significance in headache perception between child and their mothers.

Furthermore, given the simple nature of the Migraine ID questionnaire which consists of only a few targeted questions, we cannot rule out that the reported headache episodes in the last 3 months may be secondary or transient rather than primary migraines.

In addition, the simplicity of the ID Migraine allows us to observe a quantitative discrepancy in the responses between children and mothers, but more complex questionnaires are needed to clarify the qualitative aspects related to this discrepancy.

A discordant finding from the literature [10] is the nearly double prevalence of females reporting headaches in our sample, whereas previous studies suggest a more balanced male/female distribution or a slight male predominance in the pediatric population aged 7–11 years. Several factors may explain this discrepancy: (1) our sample included individuals aged 10–12 years, an age range in which prepubertal changes begin or may have already occurred, potentially leading to an increased headache prevalence among females; (2) the low prevalence of migraine, according to ID Migraine, suggests that tension-type headaches were the most represented headache type in our general population sample, which aligns with findings that tension-type headaches are generally more prevalent in the overall population [10]; and (3) the relatively small sample size may have masked an equal sex distribution, which could be more evident with a larger sample. Epidemiological studies that analyze the general population with age-specific breakdowns may provide better clarity on this aspect.

Despite these limitations, a notable observation from our review is the discrepancy in responses between mothers and children, highlighting the importance of considering both perspectives rather than relying solely on the mother's input, as is often the case in clinical practice [1].

## 5. Conclusions and Future Directions

Our study suggests that great care should be taken when gathering information about children's headaches, especially since information is often derived from at least two sources. Future research should involve much larger samples of children and both parents, utilizing questionnaires that assess not only previous pain experiences but also the subjective perception of pain. Additionally, exploring how parents perceive their own pain experiences may also influence their observations of and attention to their child's pain. For example, parents who tend to catastrophize their own pain may be more attuned and responsive to their child's pain [18].

## Figures and Tables

**Table 1:** The items of ID Migraine questionnaire are reported.

ID migraine		
Question	Answer	
• Have you had headaches in the past 3 months?	YES	NO
if YES at the first binding question continue…		
1. When you have a headache do you experience nausea (e.g., feeling of wanting to vomit) or gagging?	YES	NO
2. Does the light bother you (much more than when you do not have a headache), e.g., do you prefer to be in the dark?	YES	NO
3. Has the headache limited your ability to study, play, or do what you have to for a while (at least 2 h)?	YES	NO

*Note:* 2 or 3 YES answers to the ID.

Migraine = Possible Migraine.

**Table 2:** The main results of two samples are summarized.

Data results		
Children examined (*n*)	*n*. 68 (100%)	
Sex (*n*)	Males: *n*. 33 (48.6%)	Females: *n*. 35 (51.4%)
Headaches reported in the last 3 months (based on child or mother's ID Migraine questionnaire)	24/68 (45.7%)	
Positive for headache in the last 3 months by sex	Males: *n*. 8/33 (24.2%)	Females: *n*. 16/35 (45.7%)
Presence of 2 of 3 criteria for migraine	*n.* 2/68 (2.95%)	
Presence of 1 of 3 criteria for migraine	*n*. 11/68 (16.1%)	
Presence of 0 of 3 criteria for migraine	*n*. 11/68 (16.1%)	
Disagreeing responses between mother and child (one or more questions)	*n*. 23/68 (33.8%)	
Disagreeing responses between mother and child (regarding to occurrence of headache in the last 3 months)	*n*. 14/23 (61%)	
	Males: 5/33 (15.3%), Females: 9/35 (25.7%)	
Disagreeing responses between mother and child (none of the responses)	*n*. 9/23 (39%)	
Cohen's K index	0.45	

## Data Availability

The data used to support the findings of this study are available from the corresponding author upon reasonable request.
